# Effect of various additives on the properties of the films and coatings derived from hydroxypropyl methylcellulose—A review

**DOI:** 10.1002/fsn3.1206

**Published:** 2019-09-13

**Authors:** Reza Ghadermazi, Saeid Hamdipour, Kambiz Sadeghi, Rojin Ghadermazi, Asghar Khosrowshahi Asl

**Affiliations:** ^1^ Department of Food Science and Technology Faculty of Agriculture Urmia University Urmia Iran; ^2^ Department of Packaging College of Science and Technology Yonsei University Wonju Korea; ^3^ Department of Pharmaceutics School of Pharmacy Hamadan University of Medical Sciences Hamadan Iran

**Keywords:** additives, edible film, hydroxypropyl methylcellulose, permeability

## Abstract

Edible films and coating materials are commonly used as appropriate packaging materials to extend the shelf life of fresh food. Due to all their properties, edible film and coating materials have been received much attention. They are biodegradable, edible, and good barrier against environmental parameters; thereby, they could carry and deliver food additives protecting food quality. Hydroxypropyl methylcellulose (HPMC), a cellulose derivatives, can act as an excellent film‐forming agent for coating food produces. The aim of this study was to provide an overview of the HPMC properties and investigate the effects of various additives on its film‐forming properties, such as rheological behavior, water vapor, and gas permeability, as well as mechanical, optical, antioxidant, and antimicrobial properties, with a focus on the recent progress and outputs, which has been recently published. Hydroxypropyl methylcellulose is prone to be commonly used as an advanced film‐forming and coating materials for the sake of well miscibility with a wide range of organic and inorganic materials. However, this polymer requires further improvements regarding moisture susceptibility and thermal properties.

## 1 INTRODUCTION

Taking into account consumers' awareness about environmental issues, petroleum–plastic materials price increase and limitation in the resources, the great attempts are being conducted to provide natural and renewable resources for biodegradable packaging application (Ferreira, Nunes, Delgadillo, & Lopes‐da‐Silva, 2009). It seems that the application of edible films and coating is rather new, but such compounds have been frequently used to protect fresh products from moisture loss, wrinkle, and glossiness loss (Embuscado & Huber, 2009).

**Figure 1:**
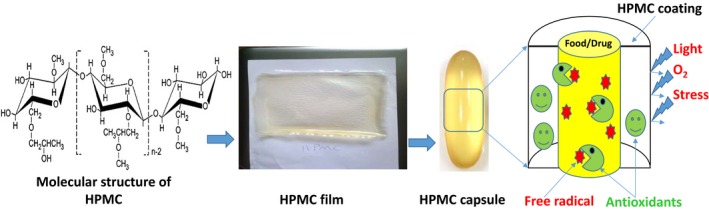
Chemical structure of hydroxypropyl methylcellulose and schematic of food and drug protected by HPMC coating containing antioxidant compounds (Source: Ghadermazi, Keramat, & Goli, 2015)

Edible films and coatings are classified into three groups, namely hydrocolloids (e.g., polysaccharides and proteins), lipids (e.g., waxes and fatty acids), and blends (blends are usually defined as a combination of two or more materials) (Bourtoom, 2008). Polysaccharides are described as good oxygen barrier materials and with stable chemical structure. However, due to the presence of hydrophilic groups, polysaccharides tend to uptake the water via surface interaction (Embuscado & Huber, 2009). Cellulose is the most abundant organic compound in the environment, which is renewable, recyclable, and biodegradable (into carbon, hydrogen, and oxygen) (Park & Chinnan, 1995). Notably, cellulose is more suitable for packing purpose as it is not a thermoplastic polymer, whereas its ester derivatives (methylcellulose [MC], hydroxypropyl methylcellulose [HPMC], hydroxypropyl cellulose [HPC], and ethyl cellulose [EC]) are biodegradable thermoplastic polymers. Hydroxypropyl methylcellulose and MC are soluble in the cold water, but after heating they form a thermally reversible and relatively hard gel by heating process at 50–80°C (Embuscado & Huber, 2009). Hydroxypropyl methylcellulose is odorless, flavorless, transparent, stable, oil‐resistant, nontoxic, and edible material with good film‐forming properties. It is a nonionic polymer with a linear structure of glucose molecules, in which its matrix is stabilized using hydrogen bonds. Methyl substitution of HPMC is performed using the substitution of free hydroxyl groups of glucose with hydroxypropyl groups (Figure 1). Such modifications tend to improve the cellulose backbone regarding viscosity, solubility, gelation, and film‐forming performance. Therefore, HPMC polymer can be used for wider applications such as drug delivery and coating (von Schantz, Schagerlöf, Nordberg Karlsson, & Ohlin, 2014).

Hydroxypropyl methylcellulose has also received GRAS‐affirmed approval by Food and Drug Administration (FDA), European Parliament and Council Directive (EU), and Joint Expert Committee on Food Additives (JECFA) (Akhtar et al., 2013; Burdock, 2007; Embuscado & Huber, 2009). Hydroxypropyl methylcellulose film properties are strongly dependent on their linear structure and molecular weight.

Accordingly, Ayrancí, Büyüktaş, and Çetin (1997) reported that with increasing molecular weight of HPMC films, WVP reduced, in which such reduction was constantly happened at HPMC films with a molecular weight higher than 41,000, whereas a similar attempt reported an increment in WVP of MC and HPC with increasing molecular weight (Park, Weller, Vergano, & Testin, 1993). In addition, Otani et al. highlighted that molecular weight is not affected WVP of HPMC films, but substitution degree can significantly change the WVP. It can be explained by changes in polarity caused by methoxyl substitution. Such changes might be related to nature of chemical structure in different cellulose derivatives because HPMC has higher methyl groups compared with MC and HPC, making HPMC films more hydrophilic (Ayrancí et al., 1997). Some reports also mention that increase in molecular weight tends to enhance the WVP of HPC and MC films (Park et al., 1993). There are various additives suitable for improving the HPMC properties such as functional performance (tensile strength or WVP) or new properties (antioxidant or antimicrobial) addition. In some cases, film‐forming compounds can be dissolved in water. In addition, some solvents (alcohols and acids) also may be used with water for increasing solubility of film‐forming compounds, but prior to application, their safety should be considered. Glycerol and sorbitol are commonly used as a plasticizer to improve flexibility. Furthermore, the emulsifier using is required for uniformly dispersion of some hydrophobic additives in the film. Wax and fat are commonly added to edible coating materials, to maintain the quality of fresh products, and prevent the wrinkling their texture. There are some other substances as nutrient compounds or food additives (such as variety of polymers, fatty acids, colors, antioxidants, and antimicrobial agents), which also can affect properties of the coating films.

The main objectives of this study are to characterize the HPMC properties and provide an overview of the effects of various additives (plasticizers, antimicrobials, and/or antioxidants) on the HPMC film performance. This review paper also considers the modifications and improvements in the HPMC film and its coating properties including the recent progress in that field.

## 2 HPMC FILM CHARACTERISTICS

Hydroxypropyl methylcellulose film is prepared using homogeneously dispersion of its powder (1%–1.5% w/w) and additives into de‐ionized water or water/ethanol solution (80°C), following by deaeration of film solution. As coating application, the food products are either sprayed on or immersed in the film solution. Moreover, cast film in the plate is dried and consequently can be used as a packaging material.

### 2.1 HPMC solution characteristics (pH, density, zeta potential, and particles size distribution of HPMC molecules)

pH is described as a basic factor for solution property control, which can effect on physicochemical properties of HPMC molecules and interactions with other molecules. The pH of pure HPMC solution without additives is 6.47–7.87. Pure HPMC can be used as film‐forming agents without any adverse interaction with foodstuff, because pure HPMC creates a relatively neutral solution (Sánchez‐González, Vargas, González‐Martínez, Chiralt, & Cháfer, 2009). The pH of the HPMC solution can be acidified with adding the natural coloring biomolecules (mixed of beetroot juice and purple carrot extract) (Akhtar et al., 2012), whereas the addition of organic acid salts can increase the pH of the HPMC solution to the alkaline condition (Valencia‐Chamorro, Pérez‐Gago, Del Río, & Palou, 2010). The antimicrobial activity of HPMC film surface can be enhanced using the acidic compounds. Further, changes in the pH of film solution tend to change the amount and type of electrical charges, which can strongly affect the interaction of HPMC with other compounds as well as its particle size or mechanical and permeability properties of HPMC films.

It has been reported that with decreasing density of HPMC, particle size and apparent viscosity of solution increase. Such changes tend to change the film thickness and reduce the strength of HPMC film, so film with higher density has the strong and stable structure than film with lower density. Hydroxypropyl methylcellulose density is approximately of 1,002.5–1,012.9 kg/m^3^. Addition of essential oils (EOs) as low‐density organic compounds into HPMC solution can be used for control and reduce the HPMC film density (Sánchez‐González et al., 2009).

The surface electrical charge plays a key role in the solution stability or sedimentation of HPMC. The pure HPMC solution has a negative charge, and its zeta potential range is −2.14 to −3.4 mV. In addition, introducing EOs into film solution can increase the total electric charge and move the solution to high negative charge region. With increasing the electrical charge of particles, the repulsive forces between particles increase, resulting in an uniform distribution of the particles in HPMC matrix. Therefore, HPMC can stabilize EOs molecules in its matrix and prevent the accumulation of EOs molecules on surface of the film. Application of emulsifier and constant homogenizing can enhance the film stability (Sánchez‐González et al., 2009). The particle charge is a critical factor of HPMC performance, particularly, regarding gelation for drug delivery and coating. The thermo‐reversible property of HPMC is dependent on its behavior in the aqueous solution. Therefore, the surface charge may control swelling, dissolution, and dispersion of HPMC in the aqueous solution (Joshi, 2011). Usually, incorporating the various EOs can increase the size of the particles among the film solution (Sánchez‐González, Chiralt, González‐Martínez, & Cháfer, 2011). Although the increase in the size of the particles in the film solution generally provides the unstable of film solution, it has been reported that the steric stabilization promoted by the particles interfacial adsorption and the high value of the particle z‐potential (significantly higher than +30 mV) ensures the stability of the emulsified system (Vargas, Albors, Chiralt, & González‐Martínez, 2009).

### 2.2 Flow behavior of HPMC film (apparent viscosity, shearing stress)

Rheology of biopolymer can control the thickness, uniformity of matrix, and film‐forming properties of film. In addition, evaluation of rheological behavior is required for processing and preparation of biopolymers such as shearing rates, filling, pumping, and spraying (García, Pinotti, Martino, & Zaritzky, 2009). The viscosity is depended on the HPMC concentration, type, and the temperature of solution. Pure HPMC solution has apparent viscosity about 15 (MPa s) at concentration of 2% at 25°C. The apparent viscosity increased with adding the plant‐based extracts (Pastor, Sánchez‐González, Cháfer, Chiralt, & González‐Martínez, 2010). The addition of various low‐density EOs was not shown significant impacts on the film solution rheology, whereas EOs increased the density coefficient and reduced viscosity. It can be explained that higher surface interaction between HPMC matrix and EO molecules can reduce the viscosity of continuous phase and enhance the solution film stability against shearing stress (Sánchez‐González, Vargas, González‐Martínez, Chiralt, & Cháfer, 2011). Introducing whey protein into HPMC film solution can reduce the viscosity and increase the density. However, there were no significant changes in the viscosity value with adding sodium dodecyl sulfate and sunflower oil into HPMC solution film (Rubilar, Zúñiga, Osorio, & Pedreschi, 2015). The effects of various additives on the HPMC solution are summarized in Table 1.

**Table 1:** Properties of HPMC films containing different additives

Additives	η^a^ (Pa s)	T (°C)	RH%	d (µm)	TS (MPa)	E %	EM (MPa)	WVP × 10^NR10^ (g/m s Pa)	OP (ml µm/m^2^ d kPa)	References
H^b^	NR	20	50	30	34	6.63	1,900	NR	NR	Möller, Grelier, Pardon, and Coma (2004)
3% SM (w/v)	NR	10	75	0.5	NR	NR	NR	0.94	NR	Villalobos et al. (2006)
2.1% SM + 0.9% SP (w/v)	NR	10	75	0.5	NR	NR	NR	1.48	NR	Villalobos et al. (2006)
H	NR	Room	65	30	28.5	9.6	NR	3.33	NR	Dogan and McHugh (2007)
3.3% MCC (3 µm)	NR	Room	69	40	37.2	4.88	NR	3.88	NR	Dogan and McHugh (2007)
H	NR	23	50	0.54	61	16	1,656	NR	159	Brindle and Krochta (2008)
75% WPI	NR	23	50	NR	7.8	47	182	NR	NR	Brindle and Krochta (2008)
63% G	NR	23	50	NR	NR	NR	NR	NR	616	Brindle and Krochta (2008)
63% GL + WPI	NR	23	50	NR	NR	NR	NR	NR	110	Brindle and Krochta (2008)
H	NR	23	30	NR	28.3	8.1	900	2.2	NR	de Moura et al. (2009)
CS/TPP (85 nm)	NR	23	30	NR	62.6	11.1	1,264	0.92	NR	de Moura et al. (2009)
H	NR	20	54.4	44	59	0.1	1,697	8	NR	Sánchez‐González et al. (2009)
2% TTO	NR	20	54.4	NR	42	0.11	956	5.2	NR	Sánchez‐González et al. (2009)
H	NR	20	50	47	63	13	2,334	4.2	NR	Pastor et al. (2010)
50% G	NR	20	50	56	16	50	421	8.8	NR	Pastor et al. (2010)
1% N	NR	20	50	70	43	26	856	4.9	NR	Pastor et al. (2010)
1% N + 50% G	NR	20	50	58	20	31	722	9.5	NR	Pastor et al. (2010)
H	NR	21	33	26	35.6	4.9	NR	4.7	NR	Bilbao‐Sáinz, Avena‐Bustillos, Wood, Williams, and McHugh (2010)
H	NR	10	58	2.5	55	7	2,550	4.6	NR	Jiménez, Fabra, Talens, and Chiralt (2010)
Starch + G	NR	25	53%	257	10	27	270	0.36	0.504	Jiménez et al. (2010)
Starch + G + CA	NR	25	53%	229	8	12	320	0.30	0.792	Jiménez et al. (2010)
33.3% G	NR	23	50	NR	15.2	70.7	274.6	20.28	232	Navarro‐Tarazaga et al. (2011)
H	NR	25	53%	NR	24.5	10.4	1,312	23.11	10.26	Jiménez et al. (2010)
50% starch	NR	25	53%	NR	13	9.4	670	23.61	0.97	Jiménez et al. (2010)
60% BW 12% + SA + 9.3% G	NR	23	50	NR	2.9	3.52	195.4	8.75	337	Navarro‐Tarazaga et al. (2011)
H	0.1272	20	53NR75	55	75	9	1,884	160	92	Atarés et al. (2011)
AA	0.121	20	53NR75	53	63	5.9	1,651	101	29.2	Atarés et al. (2011)
CA	0.130	20	53NR75	53	55	4.5	1,669	90	19.46	Atarés et al. (2011)
GO	0.143	20	53NR75	62	41	6	1,227	17	122	Atarés et al. (2011)
H	0.00441	20	54.4	1.6	56	7.9	643	7.1	NR	Sánchez‐González, Chiralt, et al. (2011)
2% BO	0.0044	20	54.4	1.1	39	2.9	444	3.1	NR	Sánchez‐González, Chiralt, et al. (2011)
2% LO	0.0043	20	54.4	5.6	40	3.9	397	4.1	NR	Sánchez‐González, Chiralt, et al. (2011)
2% TTO	0.0043	20	54.4	2.3	34	4.2	365	5.73	NR	Sánchez‐González, Chiralt, et al. (2011)
H	NR	24	30	34	28.3	8.1	900	8.9	NR	De Moura, Mattoso, and Zucolotto (2012)
H	NR	25	50	NR	77	8	NR	1.85	NR	Byun et al. (2012)
Sh + LA 20:1	NR	25	50	NR	70	7	NR	1.62	NR	Byun et al. (2012)
Sh + SA 100:1	NR	25	50	NR	50	5.5	NR	1.91	NR	Byun et al. (2012)
05% Sh	NR	25	50	NR	55	4.5	NR	1.45	NR	Byun et al. (2012)
H	NR	40	75	60	NR	NR	NR	0.18	NR	Laboulfie et al. (2013)
20% SA	NR	40	75	60	35	3	2,100	0.08	NR	Laboulfie et al. (2013)
13% PEG200	NR	40	75	60	25	6	1,600	0.03	NR	Laboulfie et al. (2013)
H	NR	NR	NR	NR	26.7	31	500	0.81	NR	Sánchez‐González, Saavedra, and Chiralt (2013)
5 Logs CFU/cm^2^ LAB	NR	5	75	NR	31.1	33	381	2.95	NR	Sánchez‐González et al. (2013)
H	NR	20	50	8.2	64.5	4.3	2,492	6.13	449,280	Akhtar et al. (2013)
4% G	NR	20	50	9.5	57.9	5.64	2,204	6.59	345,600	Akhtar et al. (2013)
4% NRC + 0.8% G	NR	20	50	4.3	39.9	8.72	1,102	16.68	43,200	Akhtar et al. (2013)
40% TP + 40% G	NR	25	75	220	NR	NR	13.9	0.37	NR	Villacrés, Flores, and Gerschenson (2014)
G	NR	25	NR	155.6	18.88	46.35	NR	0.00028	NR	Rubilar et al. (2015)
G + WPI + 0.5% oil + SDS	NR	25	NR	155.7	8.59	35.94	NR	0.00032	NR	Rubilar et al. (2015)
G + WPI + 1% oil	NR	25	NR	156.6	4.81	33.40	NR	0.00023	NR	Rubilar et al. (2015)
H + G	NR	25	50%	0.104	10.89	51.24	29.23	0.025	405.80	Klangmuang and Sothornvit (2016)
Nanoclay + G	NR	25	50%	0.118	16.34	48.19	53.86	0.026	421.44	Klangmuang and Sothornvit (2016)
Beeswax + G	NR	25	50%	0.142	12.79	56.19	30.89	0.019	538.42	Klangmuang and Sothornvit (2016)
Beeswax ‏ clay + G	NR	25	50%	0.115	10.29	46.42	39.99	0.013	454.56	Klangmuang and Sothornvit (2016)
50% G	NR	25	50	135.4	27.3	23.6	537.5	23.0	488.8	Ghadermazi et al. (2016)
20% CEO	NR	25	50	130.0	12.3	17.9	478.9	17.7	393.0	Ghadermazi et al. (2016)
20% OEO	NR	25	50	115.2	7.2	19.8	202.1	16.1	235.9	Ghadermazi et al. (2016)
20% SEO	NR	25	50	122.6	9.2	20.8	255.6	14.9	287.4	Ghadermazi et al. (2016)
H	NR	23	50	32.1	61.04	29.51	618.84	1,115,740	NR	Hay et al. (2018)
75% Na‐P	NR	23	50	32.1	50.46	21.72	708.17	568,287	NR	Hay et al. (2018)
H	NR	25	58	58	20.8	2.5	1,120.7	NR	NR	Bodini et al. (2019)
70% starch	NR	25	58	62	16.6	2.2	905.9	NR	NR	Bodini et al. (2019)
H	NR	NR	NR	500	38.1	NR	NR	0.3	1,212.9	Osman et al. (2019)
Al_2_O_3_‐NPs	NR	NR	NR	500	31.6	NR	NR	0.15	6,929	Osman et al. (2019)
SiO_2_‐NPs	NR	NR	NR	510	43.17	NR	NR	1.5	14,000	Osman et al. (2019)

Abbreviation: NR, not reported.

a η = apparent viscosity of film dispersions; T = temperature; RH = relative humidity. These environments were equilibrated before analysis. d = thickness; TS = tensile strength; E = elongation; EM = elastic modulus; WVP = water vapor permeability; OP = oxygen permeability.

b H = HPMC; SM = sorbitan monostearate; SP = sucrose palmitate; MCC = microcrystalline cellulose; WPI = whey protein isolate; G = glycerol; CS/TPP = chitosan/tripolyphosphate nanoparticles; TTO = tea tree essential oils; N = nisin; BW = beeswax; SA = stearic acid; AA = ascorbic acid; CA = citric acid; GO = ginger essential oil; BO = bergamot essential oils; LO = lemon essential oils; LA = lauric acid; SH = shellac; PEG = polyethylene glycol; LAB = lactic acid bacteria; NRC = natural red color; TP = tapioca starch; CEO = clove essential oil; OEO = oregano essential oil; SEO = sage essential oil; Na‐P = amylose–sodium palmitate inclusion complexes; NFC = TEMPO‐oxidized nano‐fibrillated cellulose; Al_2_O_3_‐NPs = aluminum oxide nanoparticles; SiO_2_‐NPs = silica oxide nanoparticles.

### 2.3 Microscopic structure of the HPMC film

**Figure 2:**
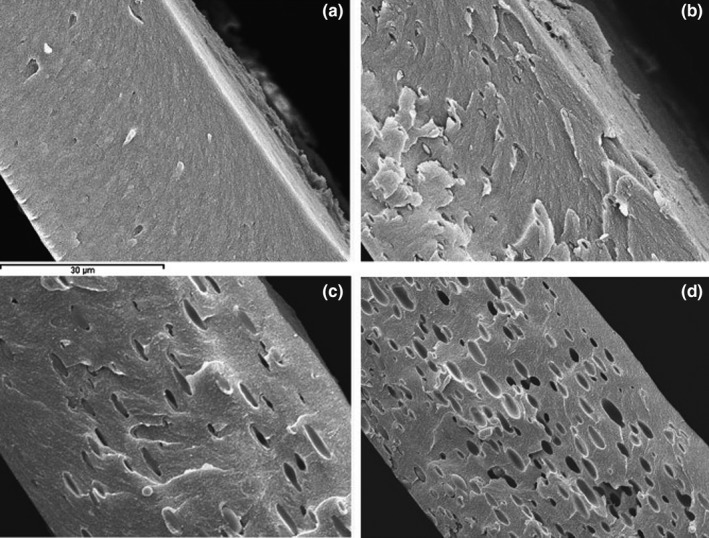
Effect of tea tree EO (TTEO) on the HPMC film morphology (a) net HPMC film, (b) HPMC + 0.5 TTEO, (c) HPMC + 1 TTEO, and (d) HPMC + 2 TTEO (Source: Sánchez‐González et al., 2009)

Pure HPMC film has a smooth surface as well as homogeneous and uniform matrix. EO addition during film production results in interaction between EO molecules and hydrophilic groups in the polymer matrix, reducing the polymer–polymer bonds and making nonuniform polymer matrix. Also, some changes in the film surface may occur during film drying process, because oil molecules tend to be accumulated on the film surface (Sánchez‐González et al., 2009). The structure of HPMC film is strongly dependent on the quality of bonds present in the film matrix, which are conducted during drying process. The nonuniform film matrix with delaminate structure is formed in the presence of various phases among polymer matrix (Figure 2).

### 2.4 HPMC film thickness

Thickness is the fundamental factor for evaluating the film performance, such as barrier properties with impact on shelf life of coated food materials. The film thickness variations are dependent on the types of incorporated materials into films matrix and preparation procedures. Because of high moisture binding capacity, HPMC film thickness could increase with the glycerol adding. Akhtar et al. (2012) reported that with the addition of water‐soluble color compounds extracted from red beet into film solution, due to combined effect of glycerol and betacyanin molecules containing lots of hydrophilic groups, the thickness of HPMC film a gradual but nonsignificant increase (Akhtar et al., 2012). According to Akhtar and Aïder (2018), incorporating glycerol (G), non‐electro‐activated whey, and electro‐activated whey into HPMC solution increased the thickness and moisture content of HPMC films. At lower concentration of non‐electro‐activated whey and electro‐activated whey (1%), there was no significant change in the film thickness, but at higher concentrations of non‐electro‐activated whey and electro‐activated whey (2%, 3% and 4%), due to film‐forming solution contains higher dry matter and less water that is evaporated during the drying process of the films, the thickness of HPMC film significantly increases.

### 2.5 HPMC film mechanical characteristics

Packaging material with good mechanical properties can protect food items inside the packaging against mechanical and physical stresses. Therefore, to extend the shelf life of food products, mechanical properties are the important properties for packaging materials. The tensile analysis is the method used for evaluating mechanical properties of the film. Tensile strength is defined as the maximum resistance of the film to breaking under tension. Elongation at break is the maximum changes in the length of the film before breakage, and the modulus of elasticity (Young's modulus) is the film stiffness value. Considering such parameters are required to determine the film‐forming capacity. Tensile strength, Young's modulus, and elongation at break of pure HPMC (5 wt%) are 28.3–64.5 MPa, 643–2,550 MPa, and 0.10%–16%, respectively (Pastor et al., 2010). Generally, HPMC has good mechanical properties and coherent structure. The incorporation of EOs can reinforce elongation at break of HPMC film, whereas decrease the tensile strength and modulus of elasticity. It implied that preparation of nonuniform polymer chain with adding EOs leads to reducing the film resistance to breaking (Ghadermazi, Keramat, & Goli, 2016; Sánchez‐González et al., 2009). Hydroxypropyl methylcellulose film containing cypress seed extract had reduced the WVP and improved the mechanical properties of HPMC film (Rhimi, Boulila, Gheribi, & Khwaldia, 2018). Hydroxypropyl methylcellulose films containing 5.0% (v/v) oregano EO nanoemulsion exhibited higher elongation at break and lower tensile strength and Young's modulus compared to pure HPMC (Lee, Garcia, Shin, & Kim, 2019). Moreover, the tensile strength of HPMC film enhanced and its elongation at break decreased with increasing the density of cellulose microcrystals, homogenizer rotation, and decreasing the size of the microcrystals particles. Hydroxypropyl methylcellulose film containing silica oxide nanoparticles (SiO_2_–NPs) had better mechanical properties compared with HPMC film containing aluminum oxide nanoparticles (Al_2_O_3_–NPs) and control HPMC films (Osman, El‐Desouky, Morsy, Aboud, & Mohamed, 2019). Such changes might be related to filler behavior of SiO_2_–NPs to occupy pores in the HPMC matrix and providing the coherent and homogenized structure in HPMC films caused by SiO_2_–NPs diffusion in HPMC matrix as well as glycerol ratio and its prevention from water evaporation. On the other hand, HPMC films containing Al_2_O_3_–NPs led to a weak structure, thereby lower mechanical properties because Al_2_O_3_–NPs cannot appropriately interact with HPMC matrix, resulting in a heterogenous dispersion. The HPMC‐based composite films containing nanoclay exhibited the higher elastic modulus and tensile strength compared with HPMC films containing Beeswax. It is implied that nanoclay in such content is sufficient to reinforce the film strength, which may be related to the presence of sufficient nanoclay in film matrix and its well dispersion (Klangmuang & Sothornvit, 2016). Hydroxypropyl methylcellulose containing zein nanoparticles showed better tensile strength, but reduced elongation at break without negative effect on the brittle nature of HPMC film. The Young's modulus increased at low concentration of zein nanoparticles but gradually decreased as the zein nanoparticles content increased (Bodini, Guimarães, Monaco‐Lourenço, & Aparecida de Carvalho, 2019).

With increasing the content of microcrystal of cellulose and homogenizing rate as well as reducing the size of microcrystals, tensile strength of HPMC film increased, whereas its tensile value reduced. The increase in tensile strength can be related to reduction in size of particles and increase in the surface areas. As such, higher surface areas make higher hydrogen bonding between polymer matrix and particles. In addition, the bigger particles interfere in gel formation during drying, resulting in further interaction between particles. Such phenomena can lead to further hydrogen bonding, thereby enhance the tensile strength of the film. Notably, the tensile strength of such composite films was almost similar to that in polyethylene terephthalate (PET) (Dogan & McHugh, 2007). It is implied that HPMC film with stronger mechanical properties could protect the products because it is crucial that film‐forming materials and coatings should be capable to protect products against mechanical stresses.

Introducing different glycerol concentrations into various polymers led to change in the tensile strength such as methylcellulose (MC) (35% glycerol), HPMC (15% glycerol), kappa carrageenan (100% glycerol), chitosan (100% glycerol), and dextrin (30% glycerol). Maximum tensile strength was related to kappa carrageenan, chitosan, HPMC, and MC, respectively. In addition, the maximum elongation at break belonged to kappa carrageenan, chitosan, and HPMC, respectively. MC and HPMC containing polyethylene glycol 400 showed the tensile strength of 60 kPa and 70 kPa, and elongation at break of 160% and 170%, respectively, which such polymers showed less impact of plasticizer on their matrix (Hong, Lee, & Son, 2005). Incorporating different plasticizers such as polyethylene glycol, glycerol, and 1,2‐propylene glycol exhibited strong effects on the HPMC and hydroxypropyl starch films properties such as providing lamellar structure and reducing tensile strength as well as increasing crystalline degree and elongation at break of pure HPMC (Zhang et al., 2018). Incorporating glycerol, non‐electro‐activated whey, and electro‐activated whey into HPMC film significantly decreased tensile strength and Young's modulus (Akhtar & Aïder, 2018). Bodini et al. (2019) reported that HPMC reduced the tensile strength of starch as an orally disintegrating film and caused the films to be less mucoadhesive. The orally disintegrating polymers are related to biopolymers that possess desirable properties for controlled‐release core compounds for drug delivery and controlled‐release active compounds (Dixit & Puthli, 2009). Choi, Singh, and Lee (2016) reported that active HPMC containing oregano and bergamot essential oils exhibited strong physical and mechanical properties.

### 2.6 Color characteristics of HPMC film

Color is an important film characteristic affecting consumer acceptability when film is applied on food as a wrapper (Klangmuang & Sothornvit, 2016). Naturally, HPMC is a transparent polymer. Incorporating specific materials into film affects on the film properties. It can be characterized using food color additives in the film production. Introducing EOs into HPMC film can enhance the light scattering, resulting in the reduction of transparency and surface glazing. It can be explained that different particles (size) exhibit different refractive index values in the polymer matrix. Due to additional reflection and light scattering caused by oil molecules, whiteness index increase by adding EOs into the polymer.

**Figure 3:**
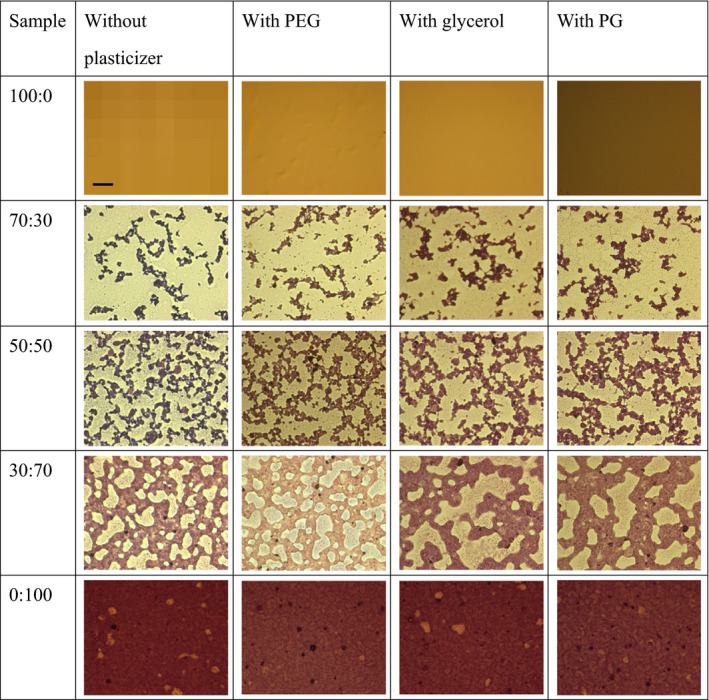
Optical microscopic images of the HPMC/hydroxypropyl starch films in the presence of different plasticizers. The scale bar equals to 80 μm, and other images have same scale (Source: Zhang et al., 2018)

Glossy surface, as a morphological parameter of film, can be obtained during drying process. A film with a smooth surface has a glossy surface, while incorporating the EOs can increase the surface roughness (Sánchez‐González et al., 2009). Pure HPMC film has a smooth and homogenous surface, but plasticizers tend to change the smooth surface to its countercurrent. Akhtar et al. (2013) found that incorporating glycerol into HPMC film relatively reduced the film transparency. Beet red pigments, depending on the added concentration, also could decrease the HPMC film transparency (Akhtar et al., 2013). Moreover, there was no significant change in color parameters by adding glycerol into the HMPC film (Imran et al., 2012). Investigation of two phases of HPMC/ hydroxypropyl starch blends is carried out using dyeing hydroxypropyl starch with iodine method for optical microscope observation. Depending of the type of plasticizer, hydroxypropyl starch is identified as relatively darker after being dyed with iodine using optical microscope observation, because of immiscibility of blends, as can be seen in Figure 3. In the 70/30 ratio of HPMC/hydroxypropyl starch, HPMC exhibited as a continuous phase, while with increasing hydroxypropyl starch content, the dark region strongly enhanced and hydroxypropyl starch replaced HPMC, which acted as a continuous phase in the blend of 30/70. In the 30/70 course as a multiphase, continuous phase can be seen as distinct starch particles in the region of the separated phase, indicating that there is an interphasic region among such blending system. It implied that HPMC and hydroxypropyl starch are relatively compatible because some glucose units have been modified using hydroxypropylene group, resulting in advanced water soluble and chemically similar polysaccharides (Zhang et al., 2018). Generally, moisture content of film cannot affect the optical properties, but optical properties are strongly dependant on the incorporating materials in the film matrix (Pastor et al., 2010).

**Figure 4:**
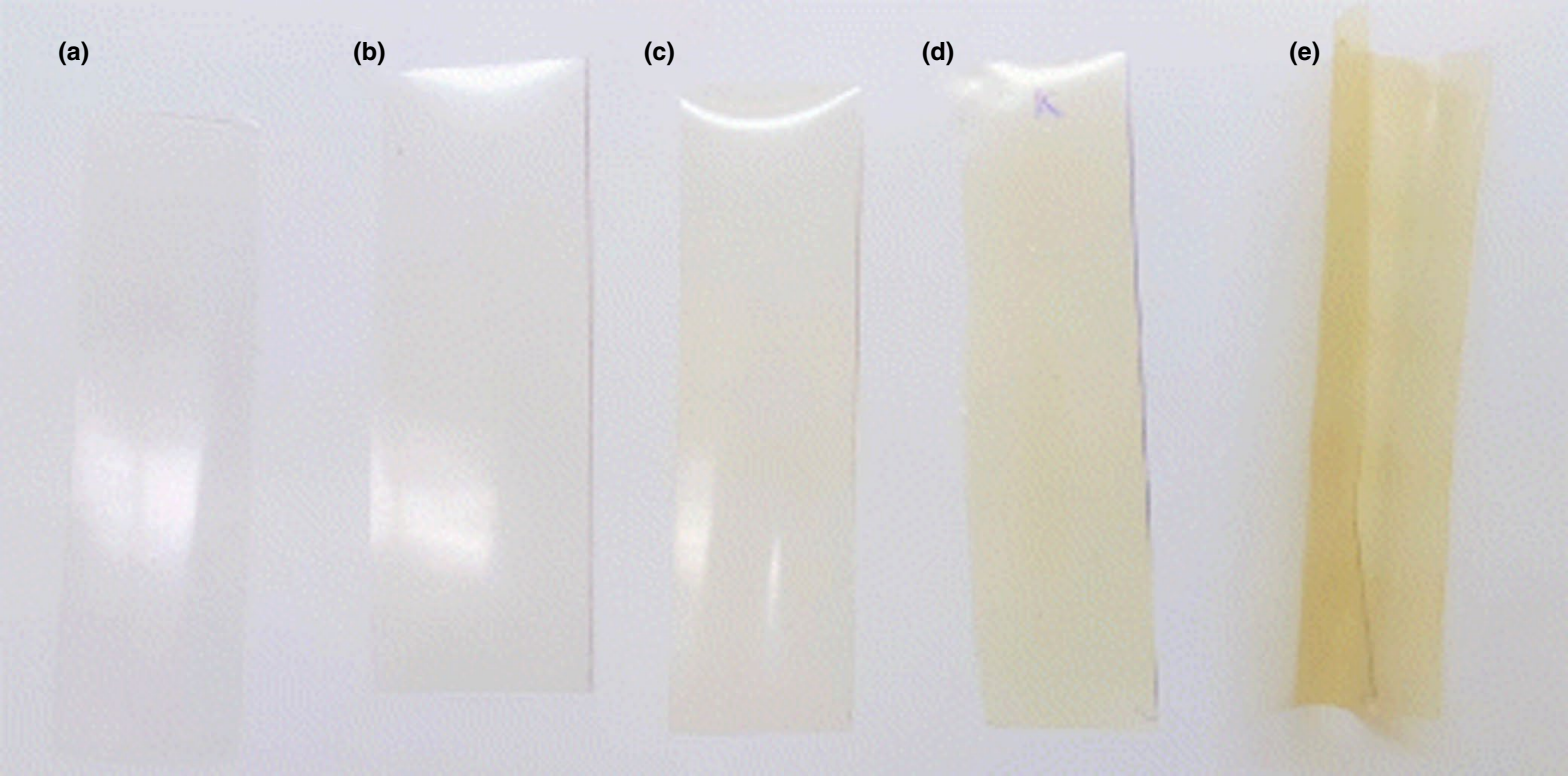
Effect of zein nanoparticles (ZNP) at different concentration of on the visual appearance of HPMC films: (a) net HPMC film, (b) HPMC + 0.018 ZNP, (c) HPMC + 0.036 ZNP, (d) HPMC + 0.155 ZNP, and HPMC + 0.268 ZNP (e) (Source: Gilbert et al., 2018)

Transparency and glossy of film significantly decrease by increasing the tea EO concentration because of strong light scattering property of the tea EO, and oil molecules can change the color of HPMC film and decrease the whiteness and transparency (Sánchez‐González et al., 2009). Additionally, incorporating other compounds, such as whey protein, surfactant, sodium dodecyl sulfate, and sunflower oil, decrease films transparency and whiteness index. Therefore, oil, surfactant, and whey protein can be described as significant transparency and color‐changing (∆E) agents. Incorporating glycerol, non‐electro‐activated whey, and electro–activated whey into HPMC film significantly increased the ΔE value (Akhtar & Aïder, 2018). It has been reported that oregano EO nanoemulsion reduced the transparency and UV transmission (Lee et al., 2019). Addition of beeswax and nanoclay into HPMC films decreased the glossiness and brightness (L*), whereas increased the yellowness (b*) and redness (a*) in which color‐changing caused by beeswax was more pronounced compared with nanoclay. Such changes are result of the natural affinity changes in film color. The whiteness of beeswax and yellowness of nanoclay naturally tend to change the color of HPMC into the new color state. In addition, there may be a binding capacity of nanoclay to the yellow color and beeswax to the white color (Klangmuang & Sothornvit, 2016). Green tea contains some active compounds such as antioxidants, minerals, and vitamins, which can be exploited in food and nutrient industry through a controlled‐release system (Cabrera, Artacho, & Giménez, 2006). In an examination, the HPMC film containing polylactic acid (PLA) nanoparticles loaded with green tea extract observed that the green tea extract slightly changed redness of HPMC film with loaded and unloaded PLA (Wrona, Cran, Nerín, & Bigger, 2017). Zein nanoparticles changed the color of HPMC film from colorless and transparent to more opaque with a yellow hue (Figure 4). It may be attributed to colloidal particles of zein nanoparticles in the HPMC film and yellow nature of zein (Gilbert, Cheng, & Jones, 2018). The nanoparticles also reduced the transparency and thickness of HPMC film (Osman et al., 2019).

### 2.7 Oxygen permeability of HPMC film

Gas permeability of HPMC film is affected by various factors, such as temperature, thickness, and environment relative humidity. Hydroxypropyl methylcellulose films tend to make crosslinks with water by increasing the relative humidity, which can increase the gas transmission rate and make the soft matrix. Hydroxypropyl methylcellulose is a strong film‐forming agent, transparent, flexible, and oxygen permeable material with appropriate sensory properties (Miller & Krochta, 1997). Beet red pigment (Betacyanins) decreases the oxygen permeability of film because free hydroxyl groups of phenolic compounds in the pigment can bond with hydroxyl groups of the HPMC film and thus make uniform and stable matrix. Moreover, during storage time, some of the phenolic compounds of pigment are hydrolyzed, resulted in smaller molecules, which filled the transmission paths among the polymer matrix. Glycerol adding into HPMC film decreases the oxygen transmission rate (Akhtar et al., 2013). Addition of EOs into HPMC film increased the gas transmission rate, but there was no enough data about it (Sánchez‐González, Chiralt, et al., 2011). Edible films and coating processing enable selective gas permeability control, which can extend the shelf life of fresh products. For example, in the internal surface of coated orange using HPMC/shellac/beeswax/glycerol containing oleic acid, the CO_2_ level was pronounced compared with O_2_ level. Such system also has better gas barrier properties compared with glazing agent commercial beeswax (polyethylene/shellac). Notably, at the end of storage, there was sufficient oxygen to prevent anaerobic microorganism activation (Contreras‐Oliva, Rojas‐Argudo, & Pérez‐Gago, 2011).

There are some important factors with impact on the fruits weight loss, such as balancing gas transmission rate, controlling level of ethanol on the fruits surface, or type and amount of fats incorporated into HPMC film. Coated fruits with HPMC film containing lipid compounds are exhibited to lower O_2_ and ethanol levels, and higher CO_2_ level on the fruit surface, because of selective gas permeability, it making modified atmosphere on surface of fruits. The reduction concentration of oxygen on surface of fruit in the lower concentration of lipids (20%) was more pronounced compared with higher lipid concentrations (60%). According to Perez‐Gago, Rojas, and DelRio (2002) with increasing ratio of HPMC to lipid, thickness of film increased due to an augment in viscosity of HPMC–lipid emulsion. Hydroxypropyl methylcellulose film containing low permeable hydrophilic compounds showed lower gas transmission rate. With increasing the lipid concentration, water vapor barrier enhanced, which delayed fruit weight loss (Perez‐Gago et al., 2002). The results were in agreement with outputs of adding the beeswax into HPMC for plums (Cv. Angeleno) preservation (Navarro‐Tarazaga, Massa, & Pérez‐Gago, 2011). These coatings reduced plum weight loss, softening, and bleeding compared to HPMC‐based coatings without beeswax, which could be related to the ability of coatings to create a modified atmosphere in the fruit.

Tangerines coated with HPMC/beeswax/shellac film containing preservatives (potassium sorbate, sodium propionate, and sodium benzoate) exhibited sufficient reduction in O_2_ level (7%) and an increase in the CO_2_ level (12%) on the fruit surface during storage. This oxygen level is not sufficient for anaerobic microorganism activation. Hydroxypropyl methylcellulose film, containing preservatives, mixture of organic acid salts, is more permeable compared with HPMC film containing a single type of salt. There is higher O_2_ level on the surface of fruits coated using mixed salts, indicating that mixed film can extend the shelf life of coated fruit (Valencia‐Chamorro et al., 2010). Klangmuang and Sothornvit (2018) investigated the quality of mango coated by active HPMC containing essential oils (ginger, plai, and fingerroot). The active HPMC coatings maintain the quality of mango during storage such as reduced weight loss, firmness loss, and color changes. Choi et al. (2016) reported that active HPMC containing oregano and bergamot essential oils exhibited strong gas permeation. Multilayer coating film (oil phase separated from hydrophilic phase) did not present significant oxygen barrier (Navarro‐Tarazaga et al., 2011). Adding glycerol to EOs decreased oxygen permeability of HPMC film. It might be attributed to filling the transmission pathways in the film matrix by smaller molecules (Ghadermazi et al., 2016).

### 2.8 Moisture content and solubility of HPMC film

**Figure 5:**
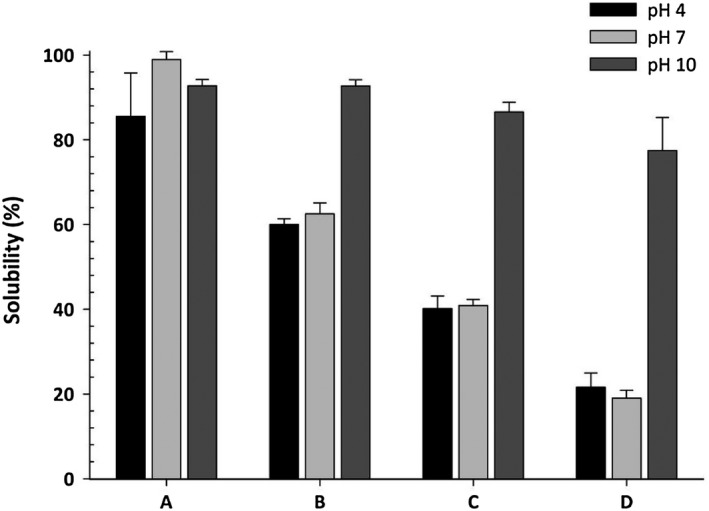
Solubility of the blending HPMC/Na‐Palm films soaked for 2 hr at pH 4, 7, and 10. A: 100% HPMC film, B: 75/25% HPMC/Na‐Palm film, C: 50/50% HPMC/Na‐Palm film, D: 25/75% HPMC/Na‐Palm film (Source: Hay et al., 2018)

Solubility is the key factor to determine the use of the film in wide applications. Films with various solubility rates can be used in wider applications. Hydroxypropyl methylcellulose is a high soluble film as a result of the presence of many hydrophilic hydroxyl groups (Figure 5). Water absorption diagram provides a comprehensive understating of water absorption capacity in any relative moisture and effects of water uptake on the softening rate and barrier properties of film. The moisture absorption curve of HPMC film is a S‐shaped curve (Sigmoid). With introducing EOs, relative humidity of film slowly increased to 6% (water activity), but its curve slop increased quickly because of high solubility in the water. On the other hand, with increasing EOs contents, moisture absorption rate of film decreased (Sánchez‐González et al., 2009).

The water absorption of HPMC film decreased with adding the surfactants (e.g., sucrose palmitate and sorbitan monostearate) because of the increasing HLB value and as hydrophilic compounds can interact with HPMC film matrix as a result of the low surface tension in the film matrix (hydrophilic–lipophilic balance). This reduction is attributed to hydrogen bonds between hydroxyl groups of surfactants and film matrix, which can reduce available active sites for bonding with water molecules (Villalobos, Hernández‐Muñoz, & Chiralt, 2006). Compounds with high hydroxyl groups can interact with active groups among the film matrix and reduce the film water absorption capacity. The HPMC film containing polyethylene glycol showed less water absorption capacity compared with polyvinyl alcohol (Okhamafe & York, 1983).

The moisture level of film is dependent on surfactants contents and its chemical structure. With increasing polarity of the surfactant, moisture of HPMC film reduced as a result of the interaction between hydrogen groups in the hydrocolloid and polar groups in the surfactant, resulting in lower interactions between polar groups and water molecules.

Hydroxypropyl methylcellulose films containing the highest surfactant concentration exhibited the highest moisture barrier (Villalobos et al., 2006). The moisture and water vapor permeability of HPMC film reduced via adding ethanolic gum. Because of further water molecules binding, film matrix swelled and became softer, resulting in reduction in polymer density and displacing the polymer chains (Pastor et al., 2010). The moisture content of HPMC film can increase with increasing the relative humidity of environment. This phenomenon firstly occurs slowly but increases gradually to reach the highest rate.

Plasticizer like glycerol also can enhance the film moisture content because of hydrogen bonding between plasticizer molecules and polymer chain, resulting in additional space between the polymer chains for water absorption. Beet red pigment added to the film tends to interact with water molecules via hydroxyl groups, which can increase the moisture content of HPMC film (Akhtar et al., 2013). As shown in Figure 5, HPMC can be dissolved in a wide range of solutions, and the addition of complexed sodium palmitate ligands (Na–palm) into HPMC film remarkably reduced its solubility, which HPMC/Na–palm became insoluble in both in the acidic (pH = 4) and pH of 7. The solubility of HPMC/Na–palm in the higher pH (10) significantly increase (Hay et al., 2018). There is no significant change in solubility of the HPMC film containing nanoparticle with pure HPMC film (Osman et al., 2019).

### 2.9 Water vapor permeability (WVP) of HPMC film

Water vapor permeability of HPMC film is being progressive because shelf life of coated food produces and preservation of dry food materials against fungi growth are strongly dependant on WVP of film. Water vapor permeability is adversely contributed to HPMC film performance. Therefore, incorporating hydrophilic compounds such as fatty acids, waxes, surfactants, and resins into the polymer are commonly used to overcome this drawback (Miller & Krochta, 1997). Hydroxypropyl methylcellulose film exhibited less WVP compared with cellophane (Villalobos et al., 2006). On the other hand, when high WVP is required, the high permeability can be an appropriate property. Incorporating hydrophobic compounds such as fats, shellacs, resins, EOs, emulsifiers, and surfactants into HPMC film reduced WVP, whereas such compounds led to an increase in brittleness and fragility. Therefore, hydrophobic compounds can be incorporated into film matrix or can be used separately on the polysaccharide and protein film. The authors were not reported any significant change in the WVP by the addition of microcrystalline cellulose particles into HPMC film (Dogan & McHugh, 2007). Water vapor permeability of film significantly varies depending on the relative humidity of environment and ambient temperature (Pastor et al., 2010).

Water vapor molecules are transmitted through film matrix in three steps: (1) Water vapor molecules are adsorbed with the film and accumulated on its surface; (2) water vapor molecules are diffused through pathways among film matrix; and (3) water vapor molecules are desorbed from the film surface (Miller & Krochta, 1997). Moreover, WVP of HPMC was decreased with increasing in the glycerol molecular weight (Ayrancí et al., 1997).

The EOs with more hydrophobic groups act as stronger water vapor barrier agents. For example, EOs extracted from bergamot and lemon reduces WVP of HPMC film approximately 50%, whereas the same concentrations of EOs extracted from tea tree decrease WVP approximately 20%. Water vapor permeability of HPMC film increase (56%–88%) with incorporating different concentrations of methanolic compounds extracted from an organic gum, in which with the methanolic compounds content increase, water vapor barrier properties enhances (Pastor et al., 2010).

The WVP of film is strongly dependant on the solubility and hydrophilicity of plasticizers or pigment compounds. The plasticizer is a low molecular weight compound, which tends to reduce the intermolecular forces between polymer chains. The plasticizer can also reinforce flexibility, elongation, and toughness of the film matrix. In general, addition of the glycerol into polymer can increase WVP. Laboulfie, Hemati, Lamure, and Diguet (2013) reported that introducing glycerol into HPMC film increased the WVP of film. Glycerol can significantly change the film properties such as reducing density and increasing WVP. Moreover, due to the presence of the polar hydroxyl groups, glycerol can reinforce the interaction between the polymer surface and the water molecules (Imran et al., 2012). Water vapor permeability of MC film could be increased up to double with incorporating 50% (w/w) glycerol (Imran, El‐Fahmy, Revol‐Junelles, & Desobry, 2010). Addition of plasticizer and beet red pigment into the film can increase WVP as a result of lower intensity of intermolecular bonds between polymer chains and reduction in the density of polymer matrix, so that the films have higher quality of mobility and additional transmission pathways among matrix.

Hydroxypropyl methylcellulose film shows higher WVP in the higher relative humidity because water molecules can interact with hydrophilic groups among film and act as plasticizer (Akhtar et al., 2013). Hydrophilic films tend to interact with water molecules, which can increase the films softness. Therefore, to evaluate the precise solubility and water vapor barrier of film, relative humidity of environment should be controlled. The amount of water vapor molecules absorbed by film matrix is attributed to the morphological and chemical structure of the HPMC film.

Water vapor permeability of HPMC film could be enhanced with increasing surfactants content. Addition of the beeswax into film reduced the WVP. The formula (WVP = 7.3e^−0.014^ *^X^*) has been presented for calculating the WVP. Accordingly, the effects of different beeswax contents on the barrier properties of the film can be precisely calculated (WVP is evaluated in mm/KPa hr m^2^, where *X* is a concentration of beeswax among film) (Navarro‐Tarazaga et al., 2011).

With incorporating 0.5% (w/w) of shellac into HPMC film, WVP decreased (11%), while this reduction increased with increasing shellac content (Byun, Ward, & Whiteside, 2012). Introducing EOs extracted from ginger into HPMC film reinforced water vapor barrier at lower temperature, comparing with higher temperature. It may be attributed to higher movement of EOs molecules toward the film surface and forming nonuniform film matrix, resulting in lower barrier properties (Atarés, Pérez‐Masiá, & Chiralt, 2011). Furthermore, with EOs content increase, WVP of HPMC and chitosan films reduces (Ghadermazi et al., 2016; Sánchez‐González, Chiralt, et al., 2011).

Water vapor permeability of film is dependent on various factors such as temperature, relative humidity, film components, and thickness. Accordingly, high permeable HPMC film is associated with its long and hydrophilic chains (Sánchez‐González et al., 2009). (2,2,6,6‐tetramethylpiperidin‐1‐yl)oxyl or (2,2,6,6‐tetramethylpiperidin‐1‐yl)oxidanyl, commonly known as TEMPO. Introducing TEMPO‐oxidized nano‐fibrillated cellulose into HPMC film improves mechanical, thermochemical, and moisture barrier properties of film (Hassan, Fadel, & Hassan, 2018). Incorporation of zein nanoparticles into HPMC film decreases the WVP of film (10%–30%) (Bodini et al., 2019). Hay et al. (2018) reported that introducing novel amylose–sodium palmitate inclusion complexes into HPMC improve the barrier properties including low water and oxygen permeability in the film without any deterioration effects on the physical properties. As result the HPMC film shows lower water uptake and moisture content as well as higher thermal stability. SiO_2_–NPs led to increasing in the WVP, CO_2_ permeability, tensile, and oxygen transmission rate (OTR). It can be explained that (a) SiO_2_–NPs as filler occupy the pore in the HPMC matrix, (b) HPMC and SiO_2_–NPs can make a coherent and uniform structure, and (c) glycerol also can reduce water evaporation (Osman et al., 2019). Incorporating glycerol, non‐electro‐activated whey, and electro‐activated whey into HPMC film significantly decreased the WVP (Akhtar & Aïder, 2018). It has been reported that oregano EO nanoemulsion reduced the WVP, indicating higher barrier properties in the HPMC film (Lee et al., 2019).

### 2.10 Thermal properties of HPMC film

Thermal properties of material can provide the information regarding degradation and decomposition of materials during heating process as well as effects of residue (degraded compounds) on the quality of materials. Therefore, thermal stability data are required for preparation, processing, and storage of materials (Rowe, 2002).

Hydroxypropyl methylcellulose particles aggregate at approximately 80°C and with decreasing temperature dissolve again. Differential scanning calorimetry (DSC) analysis is commonly used for determining the glass transition temperature (*T*_g_) of materials. The glass transition temperature is the temperature region where the polymer changes from a hard and glassy material to a soft and rubbery material. The *T*_g_ of HPMC film is 150.53°C. Because of additional transmission pathways and spaces caused by plasticizers, *T*_g_ was decreased via adding the glycerol. Incorporating phenolic Betacyanins pigment decreased *T*_g_ of HPMC film due to the presence of higher hydrogen bonds between phenolic compounds and film matrix (Akhtar et al., 2013).

### 2.11 Antioxidative properties of HPMC film

Oxidation of food can lead to off‐flavor, nutrients decomposition, or toxic material production, resulting in lower consumer–acceptability. To protect the food items from oxidation, delivering the antioxidants using biocompatible materials is still interested (Choe & Min, 2009). Edible film can be used as food antioxidants carrier and deliver them as active agents, protecting food from oxidation. Ascorbic acid, citric acid, almond oil, and EOs extracted from ginger can improve the antioxidant activity of HPMC film (Atarés et al., 2011). In addition, the shelf life of soybean oil can be significantly extended if it is packed in the HPMC films containing clove, oregano, and sage EOs (see Figure 1) (Ghadermazi et al., 2016). Hydroxypropyl methylcellulose film containing oregano EO nanoemulsion (5% v/v) presented the better antioxidant activity approximately 69% and 46% compared with pure HPMC based on the DPPH and ABTS assays, respectively (Lee et al., 2019). The maximum antioxidant activity (MMA) of cellulose ester films containing clove based on DPPH and BHA assays was 105.11% and 104.99%, respectively. Furthermore, the MMA of cellulose ester films containing cinnamon, green tea, β‐carotene, clove cinnamon, and green tea based on the BHA assay was 104.66%, 88.79%, 73.51%, 64.58%, 60.29%, and 23.42%, respectively.

The peroxide values of soybean oil packed with cellulose‐based pouches containing BHA, cinnamon oil, clove oil, and green tea extract were about 517, 484, 530, and 482 meq/kg, respectively, after 8 weeks under accelerated storage conditions (Phoopuritham, Thongngam, Yoksan, & Suppakul, 2012). Oxidation process of salmon oil packed in HPMC containing yellow and red colors (edible color) was significantly lower compared with salmon oil packed in HPMC containing blue and green colors. It may be explained that the former colors act as light block agents (dark condition) and the latter colors act as transparent film (Akhtar et al., 2010). Natural colors such as beet and carrot also act as light‐blocking agents in the HPMC film, resulting in higher antioxidant property (Akhtar et al., 2012). Rhimi et al. (2018) find that with increasing cypress seed extract concentration, water vapor barrier, opacity, and antioxidant capacity of HPMC film increased. Hydroxypropyl methylcellulose film containing 2% of cypress seed extract showed lowest oxidation degree during storage. Because of highest active phenolic/flavonoid contents, HPMC film containing 2% of cypress seed extract showed a good light barrier, resulting in a decrease in the olive oxidation (Rhimi et al., 2018).

### 2.12 Antimicrobial properties of HPMC film

Fresh food materials are prone to lose quality because of continuously physical, chemical, and microbial reactions. Such reactions may lead to foodborne disease, which can negatively impact on safety, sensory, and nutrients of foodstuffs. To prolong the shelf life of fresh food items by controlling microbial growth, protective coating using biopolymers is an effective method. The coating method can be used for agriculture products, meat, and dairy products (Cha & Chinnan, 2004). Pure HPMC film has no antimicrobial activity, but HPMC film containing preservatives (food additives) and other antimicrobial agents can possess the potent microbiostatic activity. Addition of the plant‐based extracts like propolis into HPMC film reduced the fungi growth of 2 logs (Pastor et al., 2010). Propolis naturally possesses various compounds such as phenolic compounds, essential oils, vitamin (B1, B2, B3, and B6), and organic traces (Fe or Zn) pigments, which can retard the microbial growth. Beeswax/shellac/HPMC film containing a mixture of preservatives (sodium benzoate and sodium propionate) food additives with effective inhibition of the *Penicillium digitatum* (green mold) and *Penicillium italicum* (blue mold) growth. Additionally, the HPMC film containing sodium benzoate or the HPMC film containing sodium benzoate and sodium propionate increases the zone of inhibition against green mold of 16%. This study also reported that HPMC films containing preservatives, particularly mixture of preservatives help in prolongation of fruits quality preservation with less weight loss and maintained the sensory properties (Valencia‐Chamorro et al., 2010). It has been reported that active HPMC coatings containing essential oils (ginger, plai, and fingerroot) retarded the fungal growth during storage of mango (Klangmuang & Sothornvit, 2018). Choi et al. (2016) prepared an active coating materials from HPMC containing oregano and bergamot essential oils to prolong the shelf life of plum and reduced respiration rate, ethylene production, total weight loss, and total cell count of plum during storage.

**Figure 6:**
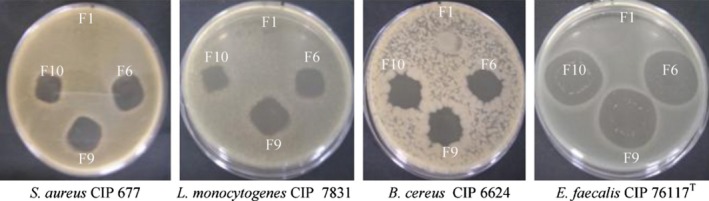
Inhibition zone of blends active films against bacteria of food origin F1 = HPMC film, F6 = HPMC + 10^4^ IU Nisaplin®, F9 = HPMC + 30% glycerol + 10^4^ IU Nisaplin,® F10 = HPMC + 50% glycerol + 10^4^ IU Nisaplin® (Source: Imran et al., 2010)

Hydroxypropyl methylcellulose films containing chitosan, tea tree oil, and bergamot EOs exhibited a remarkable inhibition growth effects against *Escherichia coli*, *Listeria monocytogenes*, and *Staphylococcus aureus* bacteria (Sánchez‐González, Chiralt, et al., 2011). Nisin is commonly incorporated into various compounds as preservative and an antimicrobial agent. Accordingly, incorporating nisin into HPMC film (10^4^ IU) presented the significant inhibition growth effects against *Listeria* spp. > *Enterococcus* spp. > *Staphylococcus* spp. > *Bacillus* spp. (Figure 6) (Imran et al., 2010). Incorporating nano‐fibrillated cellulose in the HPMC/nisin film improved the releasing rate of nisin from the HPMC film, resulting in strong biocidal activity against *S. aureus* (Hassan et al., 2018). Lee et al. (2019) reported that the HPMC film containing oregano EO nanoemulsion (5% v/v) showed good biocidal activity, particularly against *Salmonella typhimurium*. Osman et al. (2019) reported that HPMC containing Al_2_O_3_–NPs showed stronger biocidal activity against *Bacillus cereus* than *S. aureus* and *S. typhimurium*, but antimicrobial activity of HPMC containing SiO_2_–NPs against *S. typhimurium* and *B. cereus* was pronounced compared with *S. aureus*, indicating that nanoparticles with size of 80 nm at 80 ppm concentration can suppress the bacterial growth. In addition, the HPMC film containing Al_2_O_3_–NPs and SiO_2_–NPs at 80 ppm decreased the viability of foodborne pantheons in the chicken fillets during storage.

## 3 CONCLUSIONS

Hydroxypropyl methylcellulose is an edible film with strong functional properties and an applicable film‐forming agent. This film is transparent, odorless, flavorless, chemically stable, biodegradable, and nontoxic, which can extend the shelf life of fresh food products. Hydroxypropyl methylcellulose film is also a good oxygen barrier and oil‐resistant film, but HPMC film is still required further improvements regarding WVP because HPMC film is described as a high hydrophilic compound. The numerous attempts are being advanced to improve the WVP of HPMC film. Wax, beeswax, and organic EOs were added to HPMC to overcome WVP drawbacks. To improve the elasticity of HPMC film, various plasticizers such as glycerol and sorbitol are commonly used. Such compounds can enhance the elongation at break and permeability and may lead to tensile strength reduction. Antioxidant activity of HPMC film can be significantly improved using a wide range of compounds such as EOs, synthesized antioxidants, color compounds, and organic extracts. Furthermore, HPMC film is incorporated with various lipids to reduce the fruits weight loss and keep the fruits sensory properties (or quality). Hydroxypropyl methylcellulose film also can significantly extend the shelf life of fresh products by providing a modified atmosphere through the selective gas permeability without any adverse effects on the fruit sensory quality. Despite HPMC is not a strong biocidal polymer, this polymer is sufficiently miscible to be incorporated with organic and inorganic antimicrobial agents. Hydroxypropyl methylcellulose films and coatings containing antimicrobial agents have strong antimicrobial efficacy against gram‐positive/and gram‐negative bacteria as well as fungi. Hydroxypropyl methylcellulose film properties are strongly dependant on various factors such as relative humidity, temperature, thickness, production method, type, and ratio of materials incorporated into films. Among environmental parameters, relative humidity has the most significant role in the film performance and control of it is crucial for the HPMC film quality. As a functional biopolymer, HPMC is prone to be used in wider applications upon addressing the shortcomings involved with its performance. Accordingly, addition of silica‐based materials could overcome the humidity susceptibility, thermal, and mechanical imperfectness. Therefore, the upcoming attempts should be dealing with modification of physical properties of HPMC based on the expected purposes.

## CONFLICT OF INTEREST

The authors declare that they do not have any conflict of interest.

## ETHICAL APPROVAL

This study does not involve any human or animal testing.
